# Investigation of the Mechanical Properties and Microstructure of Graphene Nanoplatelet-Cement Composite

**DOI:** 10.3390/nano6110200

**Published:** 2016-11-04

**Authors:** Baomin Wang, Ruishuang Jiang, Zhenlin Wu

**Affiliations:** 1 Institute of Building Materials, School of Civil Engineering, Dalian University of Technology, Dalian 116024, China; wangbm@dlut.edu.cn; 2 School of Physics and Opto-electronic Engineering, Dalian University of Technology, Dalian 116024, China

**Keywords:** graphene nanoplatelets (GNPs), cement paste, mechanical properties, porosity, morphology

## Abstract

In this work, graphene nanoplatelets (GNPs) were dispersed uniformly in aqueous solution using methylcellulose (MC) as a dispersing agent via ultrasonic processing. Homogenous GNP suspensions were incorporated into the cement matrix to investigate the effect of GNPs on the mechanical behavior of cement paste. The optimum concentration ratio of GNPs to MC was confirmed as 1:7 by ultraviolet visible spectroscopy (UV-Vis), and the optical microscope and transmission electron microscopy (TEM) images displayed remarkable dispersing performance. The GNP–cement composite exhibited better mechanical properties with the help of surface-modified GNPs. The flexural strength of cement paste increased up to 15%–24% with 0.05 wt % GNPs (by weight of cement). Meanwhile, the compressive strength of the GNP–cement composite increased up to 3%–8%. The X-ray diffraction (XRD) and thermal analysis (TG/DTG) demonstrated that the GNPs could accelerate the degree of hydration and increase the amount of hydration products, especially at an early age. Meanwhile, the lower porosity and finer pore size distribution of GNP–cement composite were detected by mercury intrusion porosimetry (MIP). In addition, scanning electron microscope (SEM) analysis showed the introduction of GNPs could impede the development of cracks and preserve the completeness of the matrix through the plicate morphology and tortuous behavior of GNPs.

## 1. Introduction

Ordinary Portland cement (OPC) is a crucial component of concrete, which is the most popular cementitious material for architectural structures. However, OPC paste is a typically brittle material due to low tensile strength, poor flexural strength and multiple initial cracks. Traditionally, various fibers or steel bars have been used to restrict the propagation of microcracks to improve the mechanical and electrical properties of plain cement materials [1,2,3]. For the last decade, nanomaterials including nanoparticles or nanofibres have been widely applied to cement-based materials as nanofillers because of advancements in nanotechnology [4,5]. These nanosized materials could control the formation and development of nanosized cracks. Moreover, many studies have been undertaken to create carbon nanomaterial-based cement composites, including carbon nanotubes (CNTs) and carbon nanofibers (CNFs) [6,7,8,9].

As an extensively attractive carbon nanomaterial, ideally, graphene is also able to remarkably reinforce cement-based materials due to its excellent mechanical properties. The average tensile strength and Young’s modulus of graphene are 125 GPa and 1.1 TPa [10,11]. In addition, the special surface area of graphene can theoretically reach up to 2630 m^2^/g [12], which provides more potential sites for surface adsorption or other interactions between graphene and cement. In the last several years, graphene has been applied to polymers, ceramics or rubbers [13,14,15] as a reinforcing material. Meanwhile, the introduction of graphene into cementitious materials has attracted the comprehensive attention of many researchers and engineers. Gong et al. [16] found the incorporation of 0.03% graphene oxide (GO) increased the tensile and compressive strength of the cement paste by more than 40%, and the degree of hydration of GO–cement composites was promoted. Saafi et al. [17] observed that a 134% and 56% enhancement in flexural strength and flexural toughness of graphene–geopolymer composites were obtained for the addition of 0.35 wt % reduced graphene oxide (rGO). Other researchers have incorporated graphene or graphene oxide into cement-based composites as a filler material to enhance the flexural strength by 40%–60% and the compressive strength by more than 10% [18,19,20].

Graphene nanoplatelets (GNPs) consist of several graphene layers with a thickness in range of 3–100 nm [21]. Compared with single layer graphene, GNPs are not only a remarkable reinforcing material due to their morphological structure like monolayer graphene, but also low-cost, which further expands their application prospects. To date, GNPs have been extensively applied in polymeric or ceramic composites [22,23,24,25], whereas their use in cementitious materials has remained limited. Recently, Ranjbar et al. [26] reported that the compressive and flexural strength of a fly ash-based geopolymer were improved by 1.44 and 2.16 times with the help of GNPs. Peyvandi et al. [27] found that the reinforcement efficiency of different graphite nanomaterials in cementitious paste. The flexural strength of cement had various gains ranging from 27% to 73% with the addition of 0.13 wt % different GNP type and their oxide. Owing to the planer geometry and good chemical bonding with the matrix, GNPs have the ability to transfer the stress to the other positions and relieve the stress concentration in the matrix. Furthermore, GNPs may provide a larger thermal and electrical contact area due to its unique flat morphology, and thus Sedaghat et al. [28] introduced various quantities of graphene to measure the thermal diffusivity and electrical conductivity of graphene–cement composites. The electrical conductivity and thermal diffusivity of the composites were enhanced by the incorporation of graphene, and the content of needle or rod-shaped ettringite was reduced. Meanwhile, Du et al. [29] found that the GNPs significantly decreased the water penetration depth and aggressive ions ingress of cement mortar, because the layered structure of GNPs could increase tortuous paths. In a word, utilizing the outstanding properties of GNPs, the GNP-reinforced building materials can be endowed with high mechanical, electrical, thermal properties and excellent durability. However, it is a very formidable challenge to resolve the dispersion of GNPs in water and cement-based materials arising from their surface hydrophobicity and strong interlayer van der Waals forces [30].

As a traditional surfactant, methylcellulose (MC) is always applied for the dispersion of CNTs and CNFs because of its wettability, dispersibility and adhesion properties [31,32,33]. In this paper, methylcellulose (MC) was employed to dispersion GNPs in aqueous solution with the help of sonication. The ultraviolet visible spectroscopy (UV-Vis) was used to determine the optimum concentration ratio for dispersing. Moreover, the dispersing performance of GNP suspension was evaluated using optical microscope and transmission electron microscopy (TEM). Consequently, the homogenous GNP suspensions were incorporated into cement to investigate the mechanical properties of GNP–cement composites. In addition, the X-ray diffraction (XRD) and thermal analysis (TG/DTG) were used to explore the effect of GNPs on the degree of cement hydration. The pore size distribution and microstructure of GNP–cement composites were studied using mercury intrusion porosimetry (MIP) and scanning electron microscope (SEM).

## 2. Results

### 2.1. Dispersibility of GNPs in Aqueous Solution

#### 2.1.1. UV-Vis Absorbency Analysis of GNP Suspension

**Figure 1:**
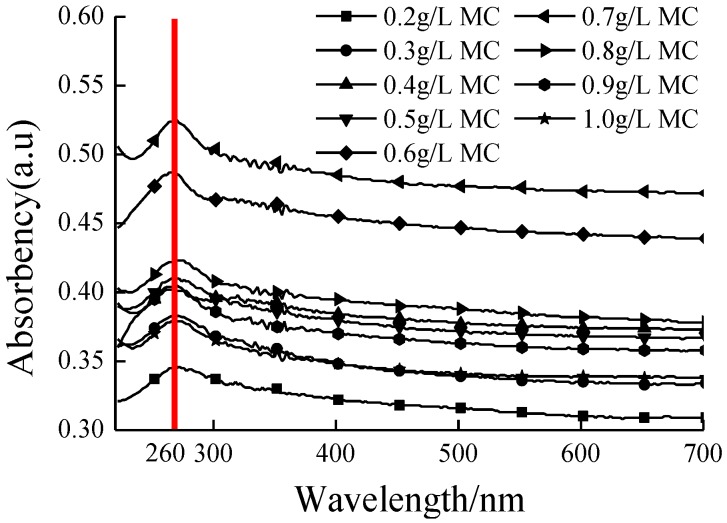
Ultraviolet visible spectroscopy (UV-Vis) absorbance spectra of graphene nanoplatelet (GNP) suspension with different methylcellulose (MC) concentrations.

**Figure 2:**
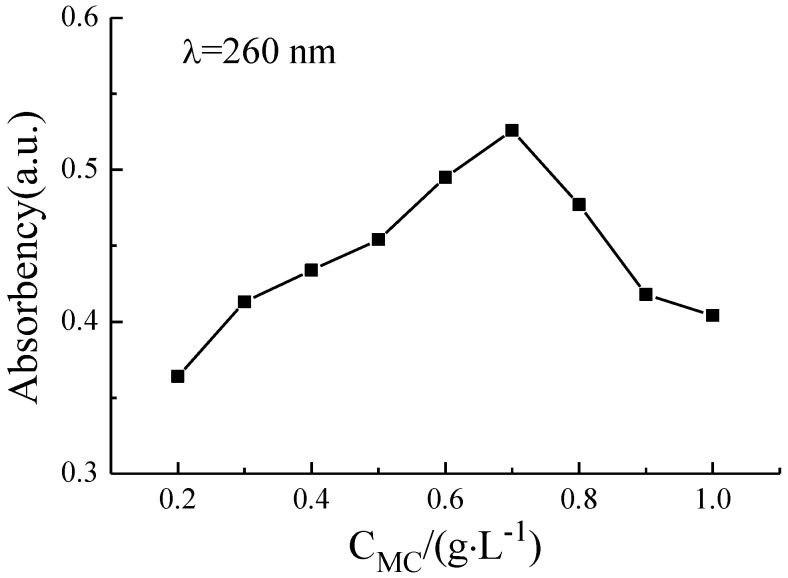
The absorbency of GNP suspension as a function of MC concentration.

The ability of MC to disperse GNPs in aqueous solution could be explored by UV-Vis absorbency spectra. According to the Lambert-Beer’s law, the higher absorbency implies that a better dispersion of GNPs in water. Figure 1 shows the absorption spectra of GNP suspensions at various MC concentrations. For all spectra, there exists an absorption peak of GNP suspension at the wavelength of 260 nm (the red line in Figure 1), which is specific absorption peak of individual GNPs. When the MC concentration is 0.7 g/L, the absorption peak of GNP suspension reaches a maximum. At the lower or higher MC concentration, the absorbency of GNP suspension is lower. In order to understand the evolution of the absorbency of GNP suspension as a function of the MC concentration, the precise absorbency of GNP suspension was measured at 260 nm (the maximum absorption wavelength). As shown in Figure 2, with an increasing in MC concentration, the absorbency of GNP suspension increases gradually, and the value of maximum absorbency is achieved at 0.7 g/L MC, which consists with the result of Figure 1. The excessive MC has a negative effect on the dispersion of GNPs. The UV-Vis absorbency analysis indicates that the optimum concentration ratio of MC to GNPs is 7:1.

#### 2.1.2. Optical Microscope and TEM Analysis of GNP Suspension

**Figure 3:**
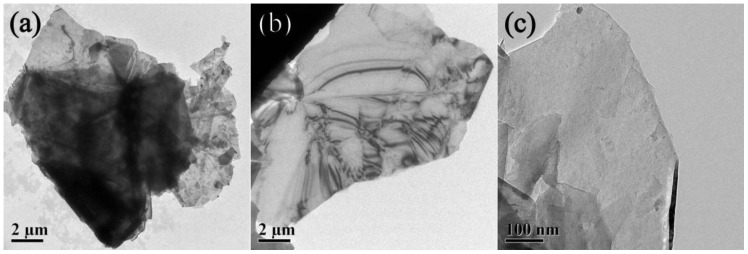
Transmission electron microscopy (TEM) images of GNP suspension: (**a**) before dispersion; (**b**,**c**) after dispersion.

The optimum dispersing status of GNPs in aqueous MC solution was employed using TEM. Figure 3a shows the overlap between layer and layer of GNPs before the addition of the MC. After dispersed using MC in combination with sonication, GNPs are highly transparent and have few wrinkles on the surface (Figure 3b). As may be noted in Figure 3c, the edges and surface of GNPs were smooth and without lager defects, which indicates that the surface treatment of GNPs using surfactant can preserve the original structure of GNPs and does not introduce other defects.

### 2.2. Workability

By conducting slump flow test, the workability is evaluated and compared between the plain cement and the GNP–cement composite, as shown in Table 1. The mean spread diameter of the plain cement was probably 89 mm, while the diameter of GNP–cement composite is merely 68 mm. The mean spread diameter of the cement paste is reduced by 30.9% with the addition of 0.05 wt % GNPs. In previous literature, there was a distinct reduction of workability when the nano additives including GO or GNTs were incorporated into cement [16,19,34]. The result could be attributed to the large specific surface area of GNPs that consumes more available water to wet their surfaces.

### 2.3. Mechanical Properties of GNP–Cement Composite

**Figure 4:**
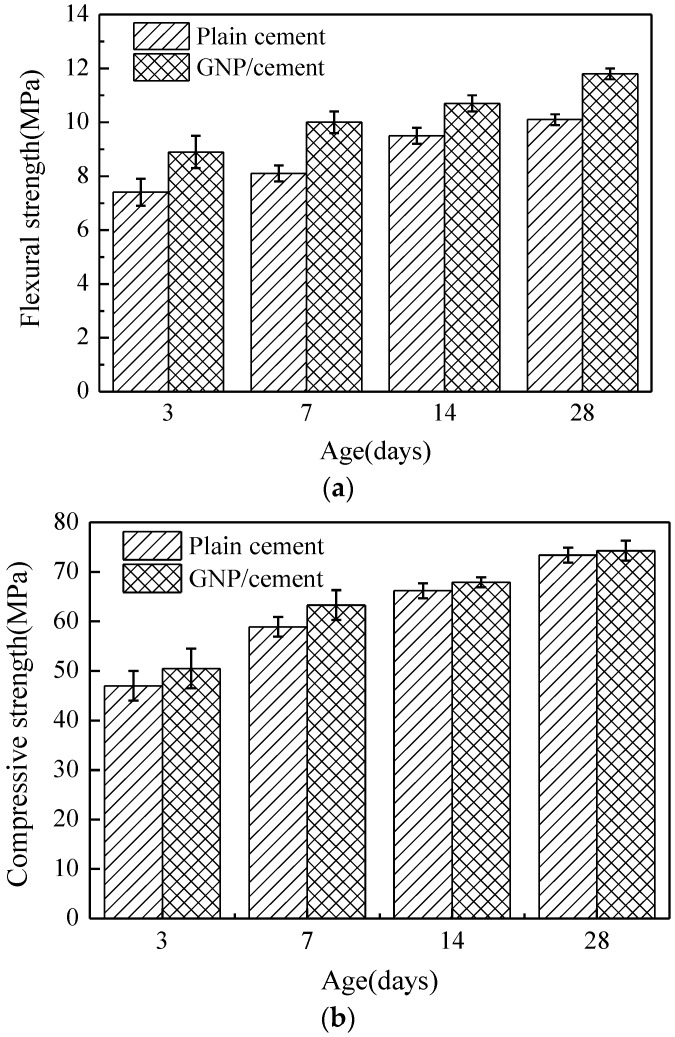
Mechanical properties of plain cement and GNP-cement composite at various ages. (**a**) Flexural strength of plain cement and GNP-cement composite at various ages. (**b**) Compressive strength of plain cement and GNP-cement composite at various ages.

The results of flexural strength and compressive strength of plain cement and GNP–cement composite at 3, 7, 14 and 28 days are shown in Figure 4. From the Figure 4, the flexural strength and compressive strength of both samples increase with reference to the ages, which is due to the ongoing hydration of cement. The results indicate that the mechanical properties of the samples reinforced with GNPs are higher than the plain cement samples at various ages. It is noted that the flexural strength of GNP–cement composite shows a more remarkable increment with respect to the compressive strength. The incorporation of GNPs enhanced the flexural strength by 15%–24% and compressive strength by 3%–8%, respectively. At the age of 7 days, the flexural strength of plain cement sample is increased by 23.5% to 10 MPa by the addition of 0.05 wt % GNPs by weight of cement. Meanwhile, the compressive strength of plain cement sample is increased by 7.5% to 63.3 MPa. However, when the age of the samples reaches 28 days, the growth rates of flexural strength and compressive strength are 16.8% and 1.3%, respectively. The introduction of GNPs may greatly enhance the strength of cement-based materials at early ages.

### 2.4. XRD Analysis

**Figure 5:**
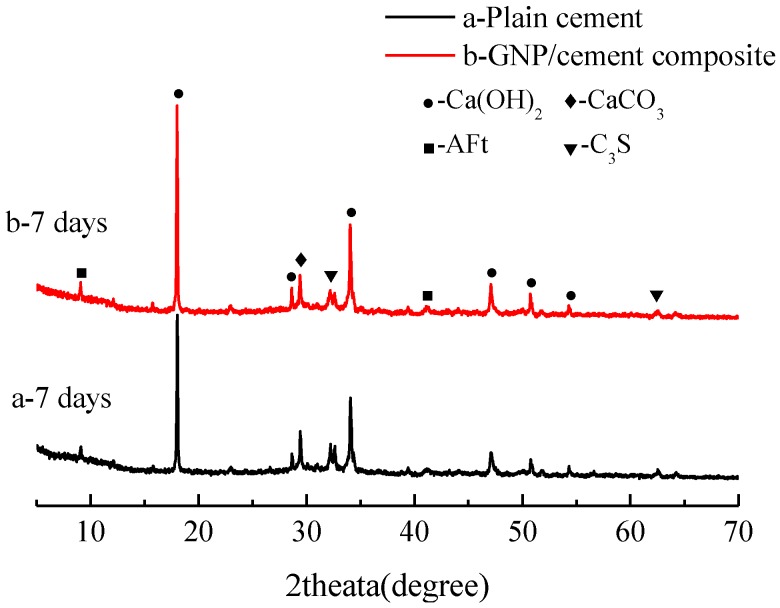
X-ray diffraction (XRD) patterns of (**a**) plain cement paste and (**b**) GNP-cement composite at the age of 7 days.

**Figure 6:**
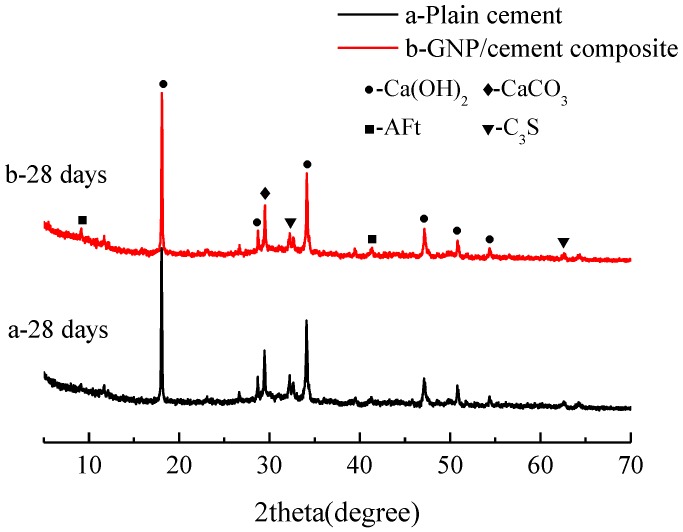
XRD patterns of (**a**) plain cement paste and (**b**) GNP-cement composite at the age of 28 days.

The effect of GNPs on the products of cement hydration can be estimated using X-ray diffraction patterns. The XRD patterns of plain cement and GNP–cement composite are shown in Figure 5 and Figure 6. After the curing period of 7 days and 28 days, several representative hydration phases have been depicted in two samples, including calcium hydroxide (Ca(OH)_2_), ettringite (AFt). Compared with plain cement, there are no new phases of the GNP–cement composite, suggesting that the addition of GNPs cannot change the type and structure of final hydration products.

Furthermore, XRD analysis can also characterize the degree of cement hydration by monitoring the formation of hydration products and the consumption of cement raw constituent qualitatively. As can be seen in Figure 5, the intensities of peaks with respect to Ca(OH)_2_ and AFt in GNP–cement composite are both higher than those in plain cement, which indicates the higher crystalline degree of Ca(OH)_2_ and AFt. Meanwhile, the intensity of unhydrated tricalcium silicate (C_3_S) in GNP–cement composite is much lower than that in plain cement, suggesting the higher hydration degree of the GNP–cement composite. The similar tendency also is observed at the age of 28 days, though the change is not very obvious in Figure 6. The XRD analysis results demonstrate that the GNPs can accelerate the hydration of cement paste, especially at the early age. Additionally, the characteristic peaks of GNPs were not detected by XRD due to the low content of GNPs.

### 2.5. Thermal (TG/DTG) Analysis

**Figure 7:**
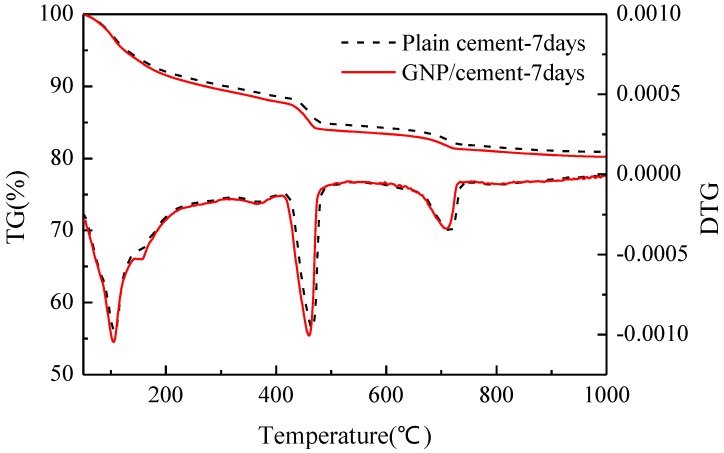
Thermal analysis (TG/DTG) curves of plain cement and GNP-cement composite at the age of 7 days.

**Figure 8:**
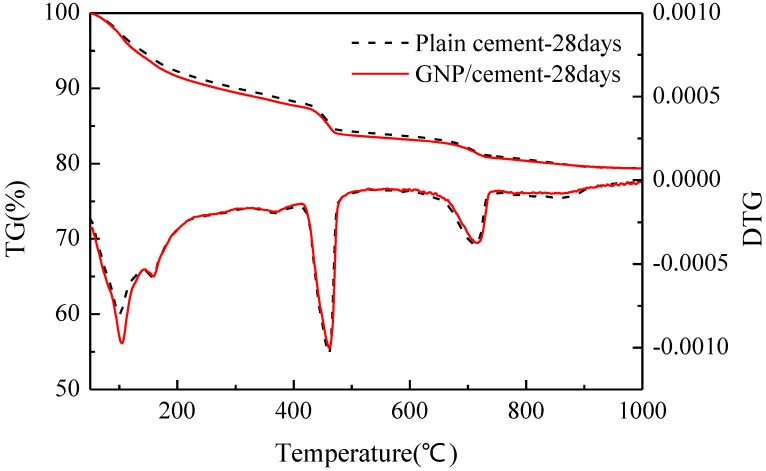
TG/DTG curves of plain cement and GNP-cement composite at the age of 28 days.

Another common method to investigate the degree of hydration is thermal analysis. The TG/DTG curves of plain cement and GNP–cement composite at the age of 7 days and 28 days have been presented in Figure 7 and Figure 8. The curves of two samples have a similar trend at various ages, demonstrating that the GNPs do not induce the formation of any other phase in accord with the results of XRD analysis. From two figures, there are four main mass losses in the DTG curve, corresponding to evaporable water (50–100 °C), portion nonevaporable water (105–200 °C), the decomposition of calcium hydroxide (400–500 °C) and calcium carbonate (650–750 °C) respectively. At the age of 7 days, the contents of amorphous phases and Ca(OH)_2_ in GNP–cement composite are higher than those in plain cement in Figure 7. However, after curing 28 days, the contents of these products is nearly equal between the GNP–cement composite and the plain cement in Figure 8. The different trend at 7 days and 28 days matches the growth rates of mechanical properties of two samples in Figure 4. The thermal analysis demonstrates further that the introduction of GNPs can accelerate the hydration process of cement.

### 2.6. Porosity and Pore Size Distribution

**Figure 9:**
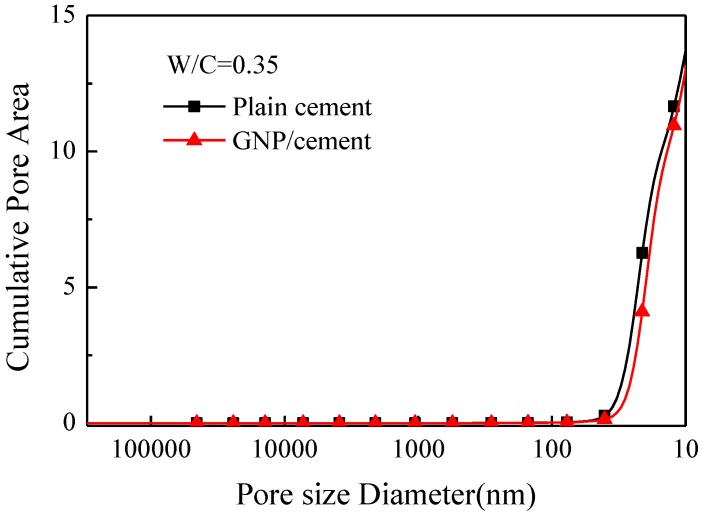
Cumulative pore area vs. pore size diameter: plain cement and GNP–cement composite at 28 days.

**Figure 10:**
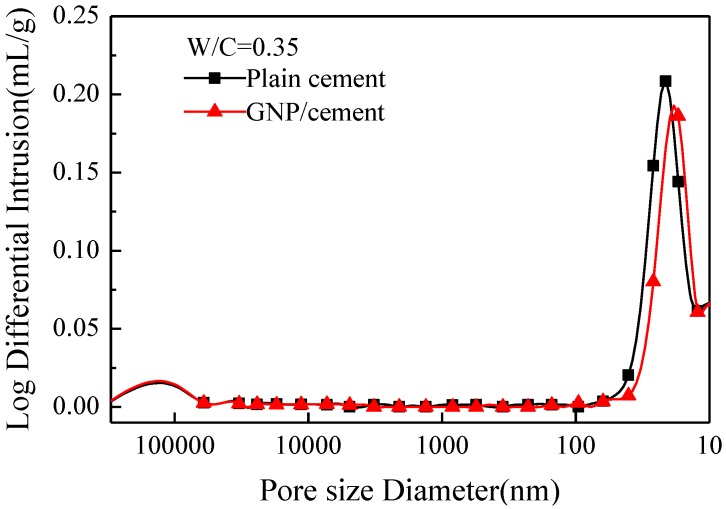
Pore size distribution of plain cement and GNP–cement composite at 28 days.

The results of porosity test for cement paste with and without GNPs at 28 days are presented in Figure 9 and Figure 10 and the total pore information is summarized in Table 2. Figure 9 shows the variation of cumulative pore area (m^2^/g) with pore size diameter (nm). The cumulative pore area of GNP–cement composite displays a decrease at various pore sizes compared with plain cement paste. The trend is similar to the change of total intrusion volume and porosity in Table 2. The total porosity of cement paste decreases from 18.35% to 17.01% by incorporating 0.05 wt % GNPs. The results indicate that the addition of GNPs can increase the compactness and decrease the porosity of the matrix.

As shown in Figure 10, the relationship between the Log differential intrusion (mL/g) and pore size diameter (nm) is observed, representing the pore size distribution of the matrix. The Log differential intrusion of plain cement paste reaches the maximum value of about 0.2088 mL/g at size diameter of 21 nm. Nevertheless, the Log differential intrusion of GNP–cement composite has a lower value of 0.1862 mL/g at size diameter of 17.1 nm. Likewise, the average pore diameter of GNP–cement composite is about 16 nm, whereas the value of plain cement paste is about 18 nm. The GNPs in cement can not only decrease the total porosity of the matrix, but also refine the pore size distribution of cement based material. Consequently, the mechanical properties of cement are enhanced by GNPs due to the improved pore characterization.

### 2.7. Microstructure

**Figure 11:**
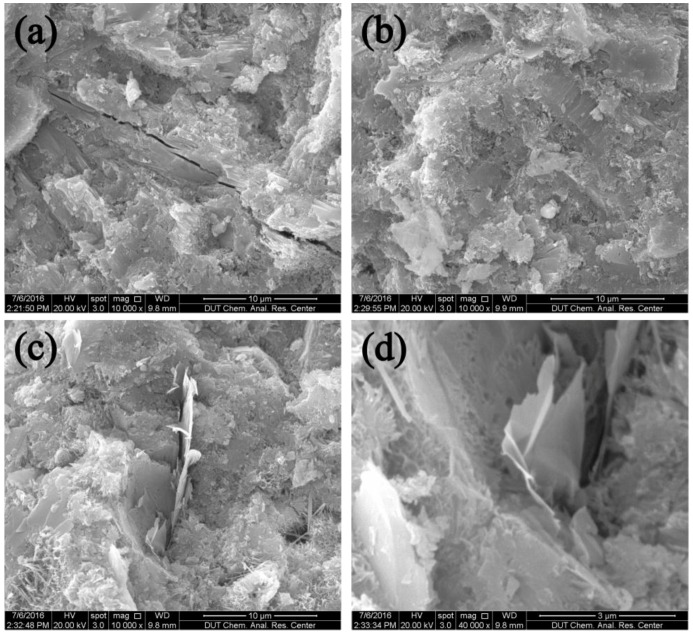
Scanning electron microscope (SEM) images of (**a**) the plain cement paste and (**b**) the GNP–cement composite at the age of 28 days. (**c**) The GNPs inserted into the hydration productions of cement. (**d**) The plicate morphology of GNPs in cement.

The comparison of microstructures between the plain cement paste and GNP–cement composite is shown in Figure 11. The plain cement paste showed a distinct crack passing straight through the whole hydration product (Figure 11a). In contrast, no obvious cracks were found and the matrix showed the denser hydration products in the GNP–cement composite (Figure 11b). Some other studies have proved that graphene can deflect or force the cracks due to its tortuous behavior [19,35]. Thus, graphene can refine the large cracks and impede the development of cracks in cement based materials.

In addition, GNPs performance in cement paste is found in Figure 11c,d. The GNPs can insert the hydration products of cement, combining well with the cement matrix (Figure 11c). As shown in Figure 11d, the GNPs have a plicate morphology and are wrapped by hydration products. This performance can prevent the crack propagation under the external load. The good interfacial interaction between GNPs and cement matrix can transfer the stress effectively, resulting in enhanced flexural strength and compressive strength of the GNP–cement composite.

## 3. Discussion

In recent years, some studies have demonstrated that the introduction of graphene can remarkably enhance the mechanical properties of ceramic and cement composites [36,37,38]. It is a key precondition for higher reinforcement of GNP–cement composite to disperse GNPs into cement matrix uniformly. In this work, the GNPs were dispersed in aqueous solution using MC as dispersant with the help of ultrasonic, and Figure 1 indicated that the optimum weight ratio of MC to GNPs was 7:1. Meanwhile, TEM test suggested a fine dispersibility of GNPs in cement matrix using the dispersing method. The workability of the cement paste presented an apparent reduction with the addition of GNPs. A lower flowability and weaker viscosity of fresh paste could affect the compactibility and the mechanical strength of GNP–cement composite. In previous reports, it was reasonable to incorporate superplasticizer to improve the workability of GNP–cement paste when a high content of GNPs is added [34]. However, the further study is needed to improve the workability of GNP–cement composite.

In addition, the hydration process of cement paste can impact flexural and compressive strength significantly. Previous research showed the effect of carbon nanomaterials on the hydration degree and products of cement paste [39,40,41]. In our work, the XRD and TG/DTG results indicate that the corporation of GNPs has a positive effect on the hydration process and does not transform the hydration products drastically. Furthermore, lower porosity and finer pore size distribution were observed in GNP–cement composite, enhancing the mechanical properties of the composite further. As a porous material, the high porosity indicates lower strength. Due to the addition of GNPs, the cement matrix becomes compacted.

**Figure 12:**
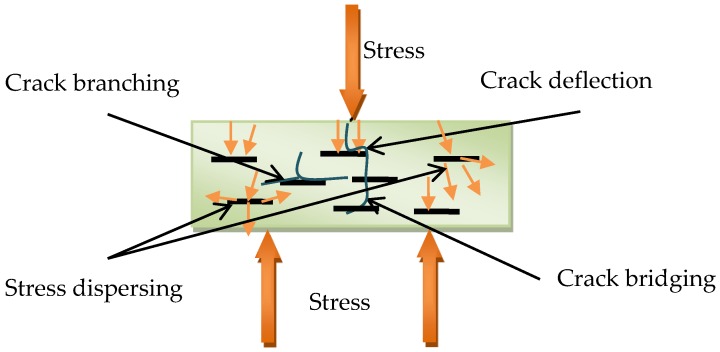
Scheme of GNP–cement composite under bending load.

The interaction between the additives and the cement matrix also has a vital impact on the properties of cement composites. In the work, there is more obvious enhancement in the flexural strength than that of the compressive strength. Some studies found that the high improvement in the flexural strength of GNP composites was due to various toughening mechanisms, including stress dispersing, crack deflection, crack bridging, and cracking branching [26,42]. As shown in SEM images, the GNPs are wrapped by hydration products, and the large aspect ratio is propitious to enhance the interfacial bond strength between GNPs and cement matrix. The strong bondability can increase the load-transfer efficiency and eliminate the destruction of stress concentration. In addition, the GNP particles provide a higher resistance to crack propagation. For the three-point bending test, the first crack form at the middle of sample. When the crack reaches the surface of GNPs, the crack develops along the interface between the GNP and cement matrix. Thus, the presence of GNPs causes crack branching or deflection, as shown in Figure 11c,d. The crack branching or deflection mechanism could increase the path of crack development, which improves the mechanical strength of cement matrix. Moreover, the crack bridging mechanism of GNPs could absorb the more energy effectively, when the composite is under external load. The whole mechanical map is presented in Figure 12. In a word, due to the high tensile strength, high Young’s modulus, and unique two-dimension morphology, GNPs could reduce the stress concentration and prevent the development of the cracks, thus enhancing the mechanical strength of the cement matrix. These results are helpful in understanding the reinforcing effect of nanofillers used for cement-based materials.

## 4. Materials and Methods

### 4.1. Materials

The cement was provided by Dalian Onoda Cement Co., Ltd. (Dalian, China), which belongs to P.O 42.5R cement (Ordinary Portland Cement) according to Chinese Standards. Its chemical composition and physical parameters are shown in Table 3 and Table 4. The GNPs (*x*-GnP-M25) were obtained from XG science, Inc. (Lansing, MI, USA). The physical properties of GNPs are presented in Table 5.

Methylcellulose (MC) as dispersant was purchased from SINOPHARM Chemical Reagent Co., Ltd. (Shenyang, China). The superplasticizer (SP) was provided by Dalian Mingyuanquan Group Co., Ltd. (Dalian, China). The tributyl phosphate (TBP) as defoamer was supplied by Tianjin Chemical Reagent Plant (Tianjin, China) in order to eliminate redundant bubbles introduced by dispersant.

### 4.2. Preparation of GNP Suspensions

The 5 mg GNPs were added into 50 mL aqueous solution with different MC concentration (ranging from 0.2 g/L to 1.0 g/L), and the suspensions were mechanically stirred for 10 min and treated for 20 min using a sonicator (operating frequency 40 KHz, power 180 W, bottom area 450 cm^2^). The treated GNP suspensions were tested to determine the best dispersion condition with regards to MC concentration.

### 4.3. GNP–Cement Composite Processing

The water to cement ratio of all samples was kept at 0.35. For GNP–cement composites, the amount of GNPs was fixed at 0.05 wt % by weight of cement. The uniform GNP suspension with weighted defoamer and dry cement were placed in an agitator kettle and the composites were mixed using a multispeed planetary mixer at low speed for 2 min and at high speed for 4 min. The mixture was then cast into 40 mm × 40 mm × 160 mm size steel molds and was vibrated for 1 min on an electric vibrator. After 24 h, all samples were demoulded and cured in 20 °C water. These samples were tested for flexural strength and compressive strength at 3 days, 7 days, 14 days and 28 days. The mix proportion of all samples is shown in Table 6.

### 4.4. Test Methods

#### 4.4.1. Characterizing the Dispersibility of GNP Suspensions

UV-Vis absorption spectra of all suspensions were measured from 200 nm to 700 nm to understand the effect of MC concentration on dispersion of GNPs, using a T6-xinshiji spectrophotometer (PERSEE Co., Beijing, China). The blank was the aqueous MC solution under the same dispersing conditions as reference solution. 

TEM images of GNP suspensions were obtained by a Tecnai G2 Spirit (FEI Co., Hillsboro, OR, USA) to represent the dispersing performance of GNP suspension. The GNP suspensions were pipetted onto a copper grid of carbon film, dried in air, and then observed in TEM.

#### 4.4.2. Slump Flow Test

**Figure 13:**
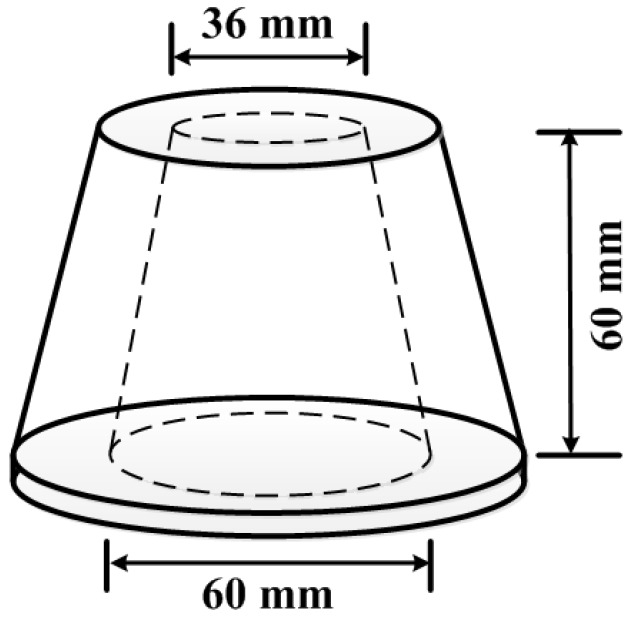
Geometry of cone mould used for slump flow test.

According to the Chinese standard “Methods for testing uniformity of concrete admixture”, the two composites were poured into a cone mould to perform the slump flow test. The dimensions of the cone mould are bottom diameter 60 mm, top diameter 36 mm, and height 60 mm, as shown in Figure 13. The cone mould was placed on a glass pane and filled with cement paste. Then, the mould was raised vertically, and the horizontal spread of the paste was recorded. The mean spread diameters of two composites were presented in Table 1. The purpose of conducting the test is to estimate the effect of GNPs on the workability of the cement.

#### 4.4.3. Mechanical Property Tests

The flexural strength was carried out by three point bending fixture on a computer controlled electrohydraulic servo universal tester (WDW-50) at a speed of 0.5 mm/min. After the bending tests, the compressive strength of each fractured fragments was conducted on a computer controlled electro-hydraulic servo universal tester (WHY-300) at a rate of 0.01 mm/min. After mechanical property tests, these fragments, including plain cement paste and GNP–cement composite, were soak in anhydrous ethanol to terminate hydration reaction.

The X-ray diffraction patterns of cement samples were recorded using an automatic diffractometer (XRD, D8 ADVANCE, Bruker AXS Co., Karlsruhe, Germany) with Cu radiation. The scan range was set for two-theta angles of 5° to 70° with a rate of 0.5 θ°/min. The hardened cement powder samples were carried out on the thermal analysis instrument (TG/DSC, Mettler Toledo Stare, Mettler Toledo Co., Zurich, Switzerland). The temperature range was from 50 °C to 1000 °C at a heating rate of 10 °C/min.

The porosity and pore size distribution of composites were detected using mercury intrusion porosimeter (MIP, AUTOPORE IV 9500, Micromertics Instrument Corp., Norcross, GA, USA), with one high-pressure station, two low-pressure stations and a maximum pressure of 33,000 psia for measurements. In addition, an environmental scanning electron microscope (SEM, QUANTA 450, FEI Co., Hillsboro, OR, USA) was performed to observe the microstructure of composites. The SEM was used in conjunction with energy dispersive spectroscopy (EDS).

## 5. Conclusions

In this paper, GNPs were treated using MC as a dispersant to improve their dispersibility in aqueous solution. The optimum MC to GNPs ratio of 7:1 by concentration was confirmed using UV-Vis absorbency, and the dispersing performance of GNPs was characterized by optical microscope and TEM. Consequently, the uniform GNP suspensions were used to reinforce the cement matrix as mixing water. The effect of GNPs on the mechanical properties and microstructure of cement based materials were investigated with the help of XRD patterns, thermal analysis, MIP, and SEM analysis.

The incorporation of 0.05% GNPs by weight of cement can enhance the flexural strength by 15%–24% and the compressive strength by 3%–8%. The XRD pattern and thermal analysis demonstrate that the degree of cement hydration has been promoted by GNPs, especially at an early age. Moreover, a more compact microstructure and finer pore size were detected in the GNP–cement composite using MIP and SEM analysis. However, more research is needed to improve the dispersibility of GNPs in cement matrix and the interfacial interaction between GNPs and the hydration products of cement. This study can provide a suitable method to investigate the two-dimensional nanomaterial for cement composites.

## Figures and Tables

**Table 1:** Workability of the mixes.

Sample	Plain Cement	GNP/Cement
Mean Spread Diameter (mm)	89 ± 3	68 ± 2

**Table 2:** Mercury intrusion porosimetry (MIP) analysis of plain cement and GNP–cement composite at 28 days.

Sample	Total Intrusion Volume/(mL/g)	Total Pore Area/(m^2^/g)	Median Pore Diameter (Volume)/nm	Median Pore Diameter (Area)/nm	Average Pore Diameter/nm	Porosity/%
Plain cement	0.0981	21.26	22	13	18	18.35
GNP/cement	0.0887	21	19	12	16	17.01

**Table 3:** Chemical composition of the cement.

CaO	SiO_2_	Al_2_O_3_	Fe_2_O_3_	SO_3_	MgO	Na_2_O
61.13	21.45	5.24	2.89	2.50	2.08	0.77

**Table 4:** Physical parameters of the cement.

Loss on Ignition/%	Setting Time/min		Special Surface Area/m^2^·kg^−1^	Compressive Strength/MPa		Flexural Strength/MPa	
	Initial Setting	Final Setting		3 days	28 days	3 days	28 days
3.50	175	235	346	6.0	8.5	30.0	53.5

**Table 5:** Physical properties of GNPs.

Products	Particle Diameters/μm	Thickness/nm	Purity/%	Electrical Conductivity/(S/m)	Special Surface Area/m^2^·g^−1^
*x*-GnP-M25	25	6–8	˃99.5	10^7^	120–150

**Table 6:** Proportion of the mixes.

Sample	Water-Cement Ratio	Mix Proportion (wt %)			
		GNPs	MC	TBP	SP
Plain cement	0.35	0	0	0.15	0.1
GNP/cement	0.35	0.05	0.35	0.15	0.1
